# The clinical features and management of pulmonary embolism at Chris Hani Baragwanath Academic Hospital 

**DOI:** 10.7196/AJTCCM.2018.v24i3.195

**Published:** 2018-09-10

**Authors:** S Meel, A Peter, C Menezes

**Affiliations:** Department of Internal Medicine, Chris Hani Baragwanath Academic Hospital and University of the Witwatersrand, Johannesburg, South Africa

**Keywords:** pulmonary embolism, South Africa

## Abstract

**Background:**

Pulmonary embolism (PE) is the most common cause of preventable deaths in hospitalised patients.

**Objectives:**

To determine the prevalence and associated features of PE at Chris Hani Baragwanath Academic Hospital (CHBAH) over a period of one year.

**Methods:**

A retrospective study was performed of patients with acute PE, as confirmed by computed tomography of the pulmonary arteries (CTPA).

**Results:**

A total of 498 CTPAs were requested during the study period. PE was confirmed in 147 (30%) of these cases. The mean age of the patients with PE was 46.8 (15.5) years. More than 40% of the patients with PE were HIV positive, of whom more than 60% had a CD4 count <200 cells/µL. Wells’ and revised Geneva scores indicated comparable clinical probability of PE. Only 15% of the patients with highrisk PE were thrombolysed, with no documented complications. There were clear contraindications for thrombolysis in only two cases, but no reasons were stated for the other cases where thrombolysis was not utilised. None of the patients had a surgical or percutaneous embolectomy. A mortality rate of 24% was found among patients diagnosed with a PE; of these, 13 (46%) presented with high-risk PE and 2 were thrombolysed. Age >40 years was the only significant predictor of mortality, as indicated by both univariate and multivariate analyses.

**Conclusion:**

PE is a common medical condition at CHBAH. The heavy infectious disease burden in the South African setting makes the diagnosis of PE challenging. Its management needs further optimisation to improve clinical outcomes.

## Background

Pulmonary embolism (PE) occurs when a blood clot embolises to the lungs from peripheral veins and causes an occlusion of the blood supply to a portion of the lung. This prevents oxygenated blood from reaching the brain and other vital organs.^[1]^

The European Society of Cardiology (ESC) guidelines have classified PE according to the estimated risk of inpatient or 30-day mortality directly related to the PE.^[2,3]^ According to this classification, a high-risk PE is characterised by haemodynamic instability, which is defined as a systolic blood pressure (SBP) <90 mmHg or a drop in blood pressure ≥40 mmHg for ≥15 minutes and which cannot be attributed to another cause (e.g. new-onset arrhythmia, hypovolaemia or sepsis).^[2,3]^ It carries a short-term mortality of 15%.^[2]^

The prevalence of PE in South Africa (SA) is unknown, but in the USA it is estimated at 600 000 cases per year.^[2]^ The prevalence among hospitalised patients over a 21-year period (1979 - 1999) in the USA was 0.4%.^[2,4]^ Mortality figures for acute PE range from 7% - 11%.^[2]^ In Sweden, an analysis of 2 356 autopsies performed in 1987 showed venous thromboembolism (VTE) in 595 cases (25%) and PE in 431 cases (18.3%). In 308 of the autopsies (13%), PE was considered to have caused or contributed to the deaths.^[2,5]^ PE is regarded as the most common cause of preventable deaths in hospitalised patients and the annual healthcare cost attributable to treatment of VTE is estimated to be more than USD1.5 billion.^[4,6]^ These studies leave no doubt that PE is a prevalent condition and is associated with a high yet preventable mortality rate.

No similar studies focusing on the clinical features and management of PE have been conducted in SA. The aim of this study was to assess the prevalence of acute PE in patients at Chris Hani Baragwanath Academic Hospital (CHBAH), as confirmed with computed tomography of the pulmonary arteries (CTPA), over a period of one year. The significance of the various clinical characteristics and the management of patients with confirmed PE were assessed.

## Methods

### Study population and sample

In this retrospective study, all CTPA reports between 1 January and 31 December 2013 were reviewed. The files of patients with confirmed acute PE were retrieved and further data were collected, including demographics, admission ward, HIV status, CD4 count, use of antiretroviral therapy (ART), D-dimer levels, mortality, and Wells’ and revised Geneva scores. The management of PE and its possible complications were also analysed. Patients with acute or chronic PE as demonstrated on CTPA were included in the study, but those with solely chronic pulmonary thromboembolic disease were not. There were no other exclusion criteria.

### Definitions

High-risk PE was characterised by haemodynamic instability as defined by the ESC guidelines.^[2,3]^ Non-high-risk PE is classified as being associated with moderate or low risk. A moderate-risk PE was diagnosed when there was right ventricle (RV) dysfunction or myocardial injury in a haemodynamically stable patient. Markers of RV dysfunction were based on echocardiographic findings of RV dilatation, hypokinesia or pressure overload, spiral computer tomography findings of RV dilatation, raised brain natriuretic peptide or N-terminal pro-brain natriuretic peptide levels, and findings of raised right heart pressure on right heart catheterisation. Positive troponin T or I was used as marker of myocardial injury. Low-risk PE is not associated with either RV dysfunction or myocardial injury.^[2]^

The ESC guidelines of 2014 do not mention the subdivision of non-high-risk PE as described here, likely to not complicate the clinically relevant distinction between high-risk and non-high-risk PE.^[3]^

### Statistical analysis

A study number was allocated to each CTPA report demonstrating a confirmed acute PE. A separate datasheet was used to correlate names with study numbers. All the captured data were recorded in a spreadsheet. The chi-square test was used to compare categorical variables. For continuous variables, Student’s *t*-test was used for comparative analysis of normally distributed variables; McNemar’s test was used to compare variables not normally distributed. Univariate and multiple logistic regression analyses were used to determine predictors of in-hospital mortality for patients with PE.

## Results

### Overall prevalence of pulmonary embolism

There were 498 requests for CTPA to screen for PE at CHBAH during the analysis period. PE was confirmed in 147 (30%) of these cases. The majority of the CTPA requests (79%) came from the medical wards.

### Demographics

The study population included 137 black patients (Table 1). The majority (78%) of the patients with PE were female. The mean age of the patients was 46.8 (15.5) years. The mean age of patients who were thrombolysed was 48.5 (14.6) years compared with a mean age of 42.6 (11.5) years in those who were not thrombolysed. This difference was not statistically significant (p=0.33).

**Table 1:** Characteristics of the study population

**Characteristics**	*n*(%)*
**Patients with confirmed PE (N=498)**	147 (29.5)
**High-risk PE (n=147)**	33 (22.4)
**Thrombolysed (n=33)**	6 (18.2)
**Age (years), mean (SD)**	46.82 (15.2)
**Systolic blood pressure (mmHg), mean (SD)**	118.2 (24.7)
**Diastolic blood pressure (mmHg), mean (SD)**	73.9 (15.5)
**Heart rate (bpm), mean (SD)**	111.5 (17.0)
**Female (n=147)**	115 (78.2)
**Ethnicity: Black African (n=147)**	137 (93.2)
**Comorbidities in patients with confirmed PE (n=147)**	
HIV-positive^†^	60 (40.8)
CD4 count <200 cells/µL (n=45)^†^	28 (62.2)
On ART (n=47)^†^	24 (51.0)
Raised body mass index^†^	24
Tuberculosis^†^	13
Malignancy^†^	7
Deep vein thrombosis^†^	16
COPD/smoker^†^	6

PE = pulmonary embolism

SD = standard deviation

bpm = beats per minute

ART = antiretroviral therapy

COPD = chronic obstructive pulmonary disease

* Unless otherwise specified

^†^ Missing data

### HIV status, CD4 counts and use of antiretroviral therapy

In this study, 60 of the 147 patients (41%) were HIV positive. The HIV status of 37 patients could not be determined, either because the files could not be found or because HIV status was not tested at the time. The CD4 counts of 45 HIV-positive patients were available and more than 60% of these patients had a CD4 count <200 cells/µL. Among the HIV-positive patients, 24 received ART; information regarding ART was missing for 13 of the HIV-positive patients.

### Serum D-dimer levels

No serum D-dimer results were available for 70 of the patients, either because the files could not be found or because the test had not been done. The average serum D-dimer level was 3.7 (3.4) mg/L (laboratory normal value range: 0.00 mg/L - 0.25 mg/L). For 4 patients, the serum D-dimer results were noted only as raised, and were therefore excluded from calculating the average. Only 1 negative result was found in the sample.

### Mortality

There were 28 deaths (24%) recorded in the sample; however, this number excludes 28 patients for whom mortality data were not available. Saddle emboli were recorded for 2 of the deceased patients. The following comorbidities were recorded, presented here in order of decreasing frequency: HIV infection; tuberculosis (TB); high body mass index (no specific values recorded); various malignancies; *Pneumocystis jirovecii* pneumonia; ischaemic heart disease; chronic obstructive pulmonary disease (COPD); and acute severe biliary pancreatitis. Two of the deaths were orthopaedic patients and 3 patients had no known comorbidities. Of the 28 deaths, 13 (46%) were patients with high-risk PE; 2 were thrombolysed. Both thrombolysed patients were HIV positive, with a saddle embolus recorded for the one and *Pneumocystis* pneumonia for the other. More than half of the deaths (54%) occurred in non-high-risk patients. The majority (79%) of the deceased patients were female.

Univariate and multivariate analyses for independent predictors of mortality were performed in a sample of 88 patients for whom all the required data were available. Univariate logistic regression analysis showed that age >40 years was the only predictive factor of mortality (odds ratio: 1.04; p=0.007). Obesity, gender, thrombolysis and HIV were not found to be statistically significant predictors of mortality based on univariate analysis. Age emerged as an independent predictor of mortality also in a multivariate model (Odds Ratio 1.06; p=0.01) when adjusted for other variables such as obesity, gender, HIV and thrombolysis (Table 2). 

**Table 2:** Multivariate analysis of predictors of mortality

**Characteristics (n=88)**	**OR (95% CI)**
Obesity	0.89 (0.22 - 3.54)
HIV	1.77 (0.55 - 5.63)
Age	1.06 (1.01 - 1.12)*
Gender	1.28 (0.37 - 4.46)
Thrombolysis	2.01 (0.31 - 12.84)

OR = odds ratio

CI = confidence interval

* p ≤ 0.05

### Wells’ and revised Geneva scores

**Fig. 1:**
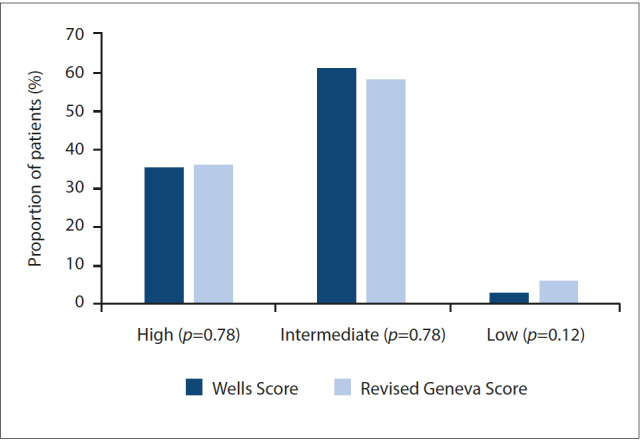
Probability of various risk categories of pulmonary embolism as indicated by Wells’ and revised Geneva scores.

Fig. 1 shows the probability for various risk categories of PE as indicated by the Wells’ and revised Geneva scores. Wells’ scores were available for 88 of the 147 patients. According to these scores, 31 patients (35%) had a high probability for PE, 54 (61%) had an intermediate probability and 3 (3%) had a low probability.

Revised Geneva scores were available for 86 of the 147 patients. According to these scores, 31 patients (36%) had a high probability for PE, 50 (58%) had an intermediate probability and 5 (6%) had a low probability.

### Thrombolysis and embolectomy

Of the 147 patients with confirmed PE, 33 (22%) met the criteria of a high-risk PE according to the ESC definition. Of these, 5 (15%) patients received thrombolysis. One patient did not meet the criteria of a high-risk PE but was thrombolysed based on echocardiographic findings of RV dysfunction. There was a 39% mortality among these 33 patients, with TB, cardiac disease and sepsis making up the majority of the comorbidities in high-risk PE patients who were not thrombolysed. There were no documented complications of thrombolysis. None of the patients had a surgical or percutaneous embolectomy and one had an inferior vena cava (IVC) filter implanted. The indication for the IVC filter was bleeding on anticoagulation in a patient with primary antiphospholipid syndrome and a confirmed popliteal deep vein thrombosis (DVT).

## Discussion

### Incidence and demographics

An earlier USA study found the prevalence of PE to be higher in black Americans than white patients.^[7]^ As blacks form the majority of the patient population at CHBAH, it is not possible to make a meaningful comparison with this study.

In a comparable study conducted in Sweden in 2006, 343 of 517 patients had confirmed VTE, with 31% of this group presenting with PE. The mean age of diagnosis was 67.6 years and 72.5 years for men and women, respectively. The most common risk factors were recent hospital admission and malignancy.^[8]^ We found a similar prevalence in our study, although the mean age of diagnosis was much lower. This is not surprising, as the majority of patients admitted at CHBAH have HIV-associated conditions and are much younger. HIV was the most common risk factor (41%), followed by obesity (16%), TB (9%) and malignancy (5%). There were, however, notable missing data.

### Interdepartmental prevalence analysis

A study to assess the trends of outcomes for PE in the USA showed that 28.8% of the patients were classified as surgical and 71% as medical.^[4]^ We found that 78% of the patients in our study were medical, possibly owing to increased vigilance among physicians with regard to the risk of PE. The low surgical referral rate is worrisome because of the obvious risk factors for PE in surgical wards. Surgery is a strong predisposing factor for VTE.^[2,3]^ However, as most studies that make up the corpus of literature on this condition are from developed countries, the impact of infectious diseases (in particular HIV infection and TB) skewing this distribution has not been explored.

### HIV infection

HIV infection is an independent risk factor for VTE, which puts the number of HIV-positive patients in this study (41%) in perspective. There is a higher incidence of VTE in HIV-positive patients with lower CD4 counts, which is related to an increasing hypercoagulable state as occurs with progressive immune suppression.^[9–11]^ Most HIV-infected patients diagnosed with PE in this study had a CD4 count <200 cells/µL, which is known to cause a higher risk of thrombosis than in patients with higher CD4 counts.^[9–11]^

Advancing age is a risk factor for thrombosis in the general population in the developed world, but the mean age of HIV-infected patients at the time of VTE is 40 years; this is 20 years younger than their non-infected counterparts.^[11]^ This finding also held true in this study, but the difference was more exaggerated. The mean age of HIV-infected patients diagnosed with PE was 24 years.

Some studies have shown that the introduction of ART has increased the incidence of VTE in HIV-positive patients, with protease inhibitors in particular being implicated.^[10,11]^ At least 40% of the HIV-positive patients in this study were on ART, although the exact regimen was not documented. As protease inhibitors form part of second-line therapy in HIV-positive patients, the increased risk for PE in these patients might also be due, in part, to their poor general state and possibly other opportunistic comorbidities.

### Thrombolysis and embolectomy

Only 5 of the 33 patients (15%) with high-risk PE and 1 patient with a moderate-risk PE were thrombolysed in this study. There were no documented complications of thrombolysis noted in any of these cases.

The management of moderate-risk PE is a subject of great debate, with no final consensus. The Moderate Pulmonary Embolism Treated with Thrombolysis (MOPETT) randomised controlled trial showed the use of lower ‘safe dose’ thrombolysis to be beneficial in lowering pulmonary hypertension after thrombolysis and 28 months thereafter. This study also showed lower mortality in the thrombolysed group and neither group had any bleeding complications.^[12]^ The much larger Pulmonary Embolism Thrombolysis (PEITHO) trial compared outcomes of patients with moderate PE who received a single-bolus dose of tenecteplase plus anticoagulation with those who received a placebo and anticoagulation. Results showed that haemodynamic decompensation was lower in the thrombolysed group at 1 week post thrombolysis but was coupled with an increased risk of stroke and extracranial bleeding.^[13]^

One patient in our study was not thrombolysed owing to recent spinal surgery and another was out of the window period for thrombolysis. There were significant comorbidities among the high-risk PE patients who did not receive thrombolysis, which could have confounded the picture of haemodynamic instability and may explain why they were not thrombolysed. Of note is that cardiac disease and COPD are associated with a poor prognosis according to the pulmonary embolism severity index for predicting 30-day mortality.^[3]^

Blood pressure fluctuations were noted for some patients who were not thrombolysed. Although there were drops of ≥40 mmHg and also SBP readings <90 mmHg, the general recovery of the SBP may have contributed to the reluctance to thrombolyse, as the patients may have been deemed to not be haemodynamically unstable despite meeting the criteria for high-risk PE as set out in the ESC guidelines.

The mortality rate was 39% among high-risk PE patients and 54% among non-high-risk patients. There were significant comorbidities in both these groups. The average age of patients who were thrombolysed was very similar to that of patients who were not thrombolysed (range 42.6 - 48.5 years), and therefore the decision regarding management was unlikely based on age.

The mortality rate among high-risk PE patients who received thrombolytic therapy has been found to be lower than in patients who had not been thrombolysed.^[14]^ The underutilisation of thrombolysis may be due to a reluctance to thrombolyse owing to potential complications and the need for close monitoring during and after thrombolysis. The presence of multiple comorbidities may also deter the treating physician from deciding on thrombolysis. There may also be a lack of awareness regarding the short- and long-term benefits of thrombolysis and the window period (14 days since onset of symptoms) during which it can be utilised.^[15]^

One patient in our sample presented with antiphospholipid syndrome and confirmed DVT and was therefore implanted with an IVC filter. The patient had active bleeding on anticoagulation. IVC filters may improve clinical outcomes of patients with a high-risk PE in addition to thrombolysis, as suggested in a large-scale retrospective USA study.^[14]^ The limitation of the USA study is that the indication for the IVC filter insertion is not mentioned. Indications for IVC filters are contentious, with only contraindication of anticoagulation or clinical failure of anticoagulation being agreed as warranting the insertion of an IVC filter.^[16]^ Although there were no other patients in this study who qualified for an IVC filter, a study focusing on VTE in general and assessing its recurrence on anticoagulation with reference to therapeutic international normalised ratios would provide a better screen for patients who may benefit from IVC filters. No surgical or percutaneous embolectomy was performed on any of the high-risk PE patients where data were available. These modalities are not considered often enough in patients where thrombolysis might be contraindicated and may prove beneficial in patients with multiple comorbidities.

### Predictors of mortality

In this study, the only variable found to be a statistically significant predictor of mortality was age (>40 years). This is not surprising, owing to the many comorbidities seen in an older population. This finding is in line with that of a study that investigated the predictors of in-hospital and long-term mortality in patients with acute PE and found older age to be a significant factor (p=0.031).^[14]^

### The Wells’ and revised Geneva scores

The Wells’ and revised Geneva scores have similar accuracy in predicting the likelihood of PE in the high-, moderate- and low-risk categories.^[2]^ These scores were not consistently documented in our sample and were calculated based on available information when missing. This study showed strong similarities between the two scoring systems and both placed the majority of patients in the moderate-risk category. Doppler ultrasounds were not always performed or the data were missing.

In this study, 7 patients had a confirmed malignancy, 1 had a premalignant lesion and 2 had suspected malignancies. Of the confirmed malignancies, the majority were haematological, which are considered to have a higher risk of VTE.^[3]^ A large proportion of the patients were HIV positive and had active TB, which are both well-known risk factors for VTE but are not included in either of these scores. Further investigation to consider whether these risk factors should be included in the scoring system for use in disease-endemic areas may be valuable.

Although the importance of clinical probability is undeniable in assessing the risk of PE, the more popular original Wells’ score includes a subjective criterion (‘alternative diagnosis less likely than PE’). This criterion carries a score of 3 and together with a non-specific criterion of tachycardia, which carries a score of 1.5 points, will already indicate the probability of PE as ‘likely’. The revised Geneva scoring system also awards 1 point to age >65 years, which, in the context of the population at CHBAH, does undermine the usefulness of the score. There are numerous clinical confounders resembling PE in the SA population, which, in turn, also increase their risk for PE. In this context, overutilisation of CTPA is deemed far less harmful than its underutilisation, considering the associated morbidity and mortality of missing the diagnosis of a PE. The importance of clinical presentation cannot be overemphasised and risk stratification scores are merely tools to improve the specificity of a certain diagnosis.

### D-dimer levels

The sensitivity of D-dimer levels as indicator of PE is well acknowledged. This was also the case in our study, with all but one patient presenting with raised D-dimer levels. The exception occurred in a patient with an underlying malignancy and recurrent DVT. His CTPA showed acute on chronic pulmonary thromboembolic disease. The negative D-dimer result may have been due to a delayed presentation of PE, which is known to notably decrease the D-dimer levels. More importantly, this case highlights the importance of clinical suspicion as being the main driver of aggressive investigation for a possible PE.

### Limitations

This was a retrospective study in which data collection relied on the manual record-keeping system at CHBAH and consequently not all the relevant information was available for all cases. This compromised the accuracy of some of the objectives of the study, especially with regard to the D-dimer levels and some risk factors. However, the proportion of the other results suggests that the findings from the available data are most likely conservative.

## Conclusion

PE is seen often in patients at CHBAH and is more common in middle-aged HIV-positive female patients. The majority of the CTPAs were requested from the medical wards. The Wells’ and revised Geneva scores are comparable in predicting the likelihood of PE. The predominant burden of infectious disease in the SA setting necessitates the need for modified probability scores that take the risk factors of HIV and TB infection into account. Thrombolysis is largely underutilised, despite being clinically indicated in high-risk PE. This may be a result of a physician’s hesitance to thrombolyse when various other comorbidities are present. In this setting, percutaneous embolectomy may prove beneficial. It is also important to heed the ESC definition of haemodynamic instability to ensure the appropriate use of thrombolysis. Ultimately more research is needed to inform the management of acute PE in developing countries with a heavy burden of infectious disease in order to optimise patient care and reduce mortality.
